# Supplementary material for: Modeling glioblastoma heterogeneity as a dynamic network of cell states
Source: Mol Syst Biol. 2021 Sep 16;17(9):e10105. doi: 10.15252/msb.202010105 (PMC8444284; doi:10.15252/msb.202010105)
Supplement: Supplementary file 6 — Source Data for Figure 5 [file MSB-17-e10105-s004.zip › Figure5A_sourcedata/GSEA_3071/nosigdb_stateIV.GseaPreranked.1622795164626/DOROSZKO_U3013MG_CLUSTER_2.html]

Details for gene set DOROSZKO\_U3013MG\_CLUSTER\_2[GSEA]

|  || Dataset | state53071 |
| Phenotype | NoPhenotypeAvailable |
| Upregulated in class | na\_neg |
| GeneSet | DOROSZKO\_U3013MG\_CLUSTER\_2 |
| Enrichment Score (ES) | -0.67346966 |
| Normalized Enrichment Score (NES) | -3.8923197 |
| Nominal p-value | 0.0 |
| FDR q-value | 0.0 |
| FWER p-Value | 0.0 |
Table: GSEA Results Summary

  

Fig 1: Enrichment plot: DOROSZKO\_U3013MG\_CLUSTER\_2      
 Profile of the Running ES Score & Positions of GeneSet Members on the Rank Ordered List

  

| PROBE | GENE SYMBOL | GENE\_TITLE | RANK IN GENE LIST | RANK METRIC SCORE | RUNNING ES | CORE ENRICHMENT || 1 | SOGA1 |  |  | 1242 | 0.453 | -0.2395 | No |
| 2 | TMSB15A |  |  | 1540 | 0.381 | -0.2948 | No |
| 3 | ACTL6A |  |  | 1789 | 0.333 | -0.3409 | No |
| 4 | PSRC1 |  |  | 1884 | 0.316 | -0.3570 | No |
| 5 | SAP30 |  |  | 1914 | 0.311 | -0.3604 | No |
| 6 | LBR |  |  | 2271 | 0.260 | -0.4281 | No |
| 7 | PTTG1 |  |  | 2470 | -0.266 | -0.4649 | No |
| 8 | LMNB1 |  |  | 2487 | -0.268 | -0.4661 | No |
| 9 | G2E3 |  |  | 2560 | -0.277 | -0.4782 | No |
| 10 | REEP4 |  |  | 2566 | -0.278 | -0.4772 | No |
| 11 | NUCB2 |  |  | 2583 | -0.280 | -0.4783 | No |
| 12 | CDKN2D |  |  | 2615 | -0.284 | -0.4823 | No |
| 13 | POP7 |  |  | 2651 | -0.290 | -0.4871 | No |
| 14 | CDC25B |  |  | 2671 | -0.292 | -0.4887 | No |
| 15 | EZH2 |  |  | 2946 | -0.324 | -0.5399 | No |
| 16 | SMC4 |  |  | 3035 | -0.337 | -0.5547 | No |
| 17 | HDGF |  |  | 3181 | -0.357 | -0.5804 | No |
| 18 | TEDC1 |  |  | 3239 | -0.366 | -0.5890 | No |
| 19 | POC1A |  |  | 3305 | -0.376 | -0.5990 | No |
| 20 | MXD3 |  |  | 3316 | -0.377 | -0.5982 | No |
| 21 | HIRIP3 |  |  | 3328 | -0.379 | -0.5976 | No |
| 22 | ILF2 |  |  | 3347 | -0.381 | -0.5984 | No |
| 23 | MNS1 |  |  | 3455 | -0.401 | -0.6164 | No |
| 24 | TROAP |  |  | 3621 | -0.432 | -0.6456 | No |
| 25 | CKAP2 |  |  | 3633 | -0.433 | -0.6446 | No |
| 26 | KNSTRN |  |  | 3646 | -0.435 | -0.6438 | No |
| 27 | PGP |  |  | 3725 | -0.450 | -0.6558 | No |
| 28 | CBR3 |  |  | 3745 | -0.454 | -0.6563 | No |
| 29 | KIF20A |  |  | 3769 | -0.459 | -0.6575 | No |
| 30 | LMNB2 |  |  | 3817 | -0.469 | -0.6633 | No |
| 31 | KIF22 |  |  | 3832 | -0.472 | -0.6626 | No |
| 32 | GPSM2 |  |  | 3841 | -0.474 | -0.6608 | No |
| 33 | ECT2 |  |  | 3902 | -0.487 | -0.6690 | No |
| 34 | MIS18BP1 |  |  | 3913 | -0.490 | -0.6674 | No |
| 35 | CDC27 |  |  | 3918 | -0.491 | -0.6646 | No |
| 36 | PTGES3 |  |  | 3953 | -0.498 | -0.6677 | No |
| 37 | CIP2A |  |  | 3968 | -0.502 | -0.6668 | No |
| 38 | CENPF |  |  | 4003 | -0.511 | -0.6698 | Yes |
| 39 | ARHGAP11A |  |  | 4007 | -0.513 | -0.6667 | Yes |
| 40 | YWHAH |  |  | 4032 | -0.521 | -0.6676 | Yes |
| 41 | PHF19 |  |  | 4041 | -0.523 | -0.6654 | Yes |
| 42 | NCAPD2 |  |  | 4055 | -0.526 | -0.6642 | Yes |
| 43 | ITGB3BP |  |  | 4071 | -0.531 | -0.6633 | Yes |
| 44 | ZWILCH |  |  | 4082 | -0.534 | -0.6614 | Yes |
| 45 | NEK2 |  |  | 4086 | -0.536 | -0.6581 | Yes |
| 46 | CCNB2 |  |  | 4103 | -0.542 | -0.6574 | Yes |
| 47 | CACYBP |  |  | 4109 | -0.542 | -0.6544 | Yes |
| 48 | BCL2L12 |  |  | 4146 | -0.553 | -0.6575 | Yes |
| 49 | FOXM1 |  |  | 4158 | -0.556 | -0.6556 | Yes |
| 50 | CENPQ |  |  | 4190 | -0.564 | -0.6576 | Yes |
| 51 | SPC24 |  |  | 4194 | -0.565 | -0.6541 | Yes |
| 52 | PLK1 |  |  | 4205 | -0.569 | -0.6520 | Yes |
| 53 | ANP32B |  |  | 4235 | -0.576 | -0.6535 | Yes |
| 54 | HMGB2 |  |  | 4243 | -0.578 | -0.6507 | Yes |
| 55 | BUB1 |  |  | 4246 | -0.578 | -0.6470 | Yes |
| 56 | BUB3 |  |  | 4249 | -0.580 | -0.6432 | Yes |
| 57 | MAGOH |  |  | 4279 | -0.592 | -0.6446 | Yes |
| 58 | SPAG5 |  |  | 4290 | -0.595 | -0.6423 | Yes |
| 59 | EXOSC8 |  |  | 4293 | -0.596 | -0.6384 | Yes |
| 60 | KIF15 |  |  | 4324 | -0.603 | -0.6399 | Yes |
| 61 | ASF1B |  |  | 4328 | -0.606 | -0.6361 | Yes |
| 62 | SGO1 |  |  | 4339 | -0.611 | -0.6337 | Yes |
| 63 | KPNA2 |  |  | 4361 | -0.621 | -0.6333 | Yes |
| 64 | KNL1 |  |  | 4367 | -0.624 | -0.6298 | Yes |
| 65 | ALYREF |  |  | 4406 | -0.638 | -0.6326 | Yes |
| 66 | RHEB |  |  | 4416 | -0.641 | -0.6297 | Yes |
| 67 | KIF14 |  |  | 4424 | -0.643 | -0.6265 | Yes |
| 68 | SAC3D1 |  |  | 4462 | -0.660 | -0.6290 | Yes |
| 69 | DDX39A |  |  | 4467 | -0.661 | -0.6250 | Yes |
| 70 | SHCBP1 |  |  | 4473 | -0.667 | -0.6212 | Yes |
| 71 | RPA3 |  |  | 4482 | -0.670 | -0.6179 | Yes |
| 72 | BUB1B |  |  | 4483 | -0.670 | -0.6131 | Yes |
| 73 | MASTL |  |  | 4490 | -0.673 | -0.6094 | Yes |
| 74 | H2AFX |  |  | 4495 | -0.677 | -0.6053 | Yes |
| 75 | DHFR |  |  | 4517 | -0.688 | -0.6045 | Yes |
| 76 | MND1 |  |  | 4538 | -0.699 | -0.6033 | Yes |
| 77 | DTYMK |  |  | 4566 | -0.715 | -0.6035 | Yes |
| 78 | NCAPG |  |  | 4568 | -0.716 | -0.5985 | Yes |
| 79 | DBF4 |  |  | 4570 | -0.717 | -0.5935 | Yes |
| 80 | TUBB4B |  |  | 4575 | -0.722 | -0.5891 | Yes |
| 81 | PBK |  |  | 4593 | -0.730 | -0.5872 | Yes |
| 82 | CKS2 |  |  | 4600 | -0.733 | -0.5831 | Yes |
| 83 | SMTN |  |  | 4603 | -0.734 | -0.5782 | Yes |
| 84 | SPDL1 |  |  | 4617 | -0.741 | -0.5754 | Yes |
| 85 | BIRC5 |  |  | 4618 | -0.745 | -0.5700 | Yes |
| 86 | CKLF |  |  | 4619 | -0.746 | -0.5647 | Yes |
| 87 | RACGAP1 |  |  | 4623 | -0.748 | -0.5599 | Yes |
| 88 | PARPBP |  |  | 4625 | -0.749 | -0.5547 | Yes |
| 89 | NCAPD3 |  |  | 4640 | -0.760 | -0.5519 | Yes |
| 90 | TTK |  |  | 4644 | -0.762 | -0.5470 | Yes |
| 91 | USP1 |  |  | 4649 | -0.763 | -0.5423 | Yes |
| 92 | SGO2 |  |  | 4650 | -0.764 | -0.5368 | Yes |
| 93 | NUSAP1 |  |  | 4654 | -0.765 | -0.5319 | Yes |
| 94 | FANCI |  |  | 4665 | -0.772 | -0.5283 | Yes |
| 95 | CENPH |  |  | 4679 | -0.786 | -0.5252 | Yes |
| 96 | CENPA |  |  | 4683 | -0.788 | -0.5201 | Yes |
| 97 | FAM83D |  |  | 4687 | -0.788 | -0.5150 | Yes |
| 98 | TRIP13 |  |  | 4721 | -0.808 | -0.5157 | Yes |
| 99 | GTSE1 |  |  | 4722 | -0.808 | -0.5098 | Yes |
| 100 | KIF4A |  |  | 4727 | -0.812 | -0.5048 | Yes |
| 101 | CDCA2 |  |  | 4732 | -0.816 | -0.4997 | Yes |
| 102 | KIF20B |  |  | 4745 | -0.825 | -0.4961 | Yes |
| 103 | CDCA8 |  |  | 4747 | -0.827 | -0.4903 | Yes |
| 104 | CENPE |  |  | 4757 | -0.832 | -0.4861 | Yes |
| 105 | MELK |  |  | 4758 | -0.835 | -0.4801 | Yes |
| 106 | KIF11 |  |  | 4760 | -0.835 | -0.4743 | Yes |
| 107 | C21orf58 |  |  | 4767 | -0.838 | -0.4694 | Yes |
| 108 | CKAP2L |  |  | 4769 | -0.839 | -0.4636 | Yes |
| 109 | NUF2 |  |  | 4782 | -0.847 | -0.4598 | Yes |
| 110 | ASPM |  |  | 4789 | -0.851 | -0.4549 | Yes |
| 111 | TMPO |  |  | 4790 | -0.851 | -0.4487 | Yes |
| 112 | CENPN |  |  | 4796 | -0.858 | -0.4435 | Yes |
| 113 | KIF23 |  |  | 4825 | -0.891 | -0.4426 | Yes |
| 114 | MKI67 |  |  | 4836 | -0.902 | -0.4381 | Yes |
| 115 | NCAPH |  |  | 4842 | -0.908 | -0.4325 | Yes |
| 116 | KIFC1 |  |  | 4844 | -0.911 | -0.4261 | Yes |
| 117 | PRC1 |  |  | 4846 | -0.912 | -0.4198 | Yes |
| 118 | SMC2 |  |  | 4847 | -0.912 | -0.4132 | Yes |
| 119 | DLGAP5 |  |  | 4857 | -0.922 | -0.4083 | Yes |
| 120 | ORC6 |  |  | 4865 | -0.931 | -0.4030 | Yes |
| 121 | PRR11 |  |  | 4868 | -0.934 | -0.3967 | Yes |
| 122 | GMNN |  |  | 4873 | -0.939 | -0.3907 | Yes |
| 123 | CDCA3 |  |  | 4874 | -0.940 | -0.3839 | Yes |
| 124 | CENPM |  |  | 4881 | -0.947 | -0.3783 | Yes |
| 125 | RFC3 |  |  | 4887 | -0.954 | -0.3724 | Yes |
| 126 | TACC3 |  |  | 4900 | -0.966 | -0.3678 | Yes |
| 127 | RTKN2 |  |  | 4904 | -0.970 | -0.3614 | Yes |
| 128 | AURKA |  |  | 4906 | -0.973 | -0.3546 | Yes |
| 129 | SKA3 |  |  | 4914 | -0.981 | -0.3489 | Yes |
| 130 | FAM111A |  |  | 4915 | -0.981 | -0.3418 | Yes |
| 131 | DNAJC9 |  |  | 4916 | -0.982 | -0.3347 | Yes |
| 132 | PSMC3IP |  |  | 4933 | -0.997 | -0.3307 | Yes |
| 133 | ATAD5 |  |  | 4934 | -0.998 | -0.3235 | Yes |
| 134 | BRCA1 |  |  | 4938 | -1.002 | -0.3169 | Yes |
| 135 | CCNB1 |  |  | 4962 | -1.032 | -0.3139 | Yes |
| 136 | CEP55 |  |  | 4972 | -1.047 | -0.3082 | Yes |
| 137 | CENPW |  |  | 4978 | -1.056 | -0.3015 | Yes |
| 138 | ATAD2 |  |  | 4980 | -1.058 | -0.2941 | Yes |
| 139 | RRM1 |  |  | 4990 | -1.074 | -0.2882 | Yes |
| 140 | HMMR |  |  | 4993 | -1.080 | -0.2808 | Yes |
| 141 | UBE2T |  |  | 5000 | -1.091 | -0.2741 | Yes |
| 142 | MAD2L1 |  |  | 5007 | -1.105 | -0.2673 | Yes |
| 143 | SPC25 |  |  | 5012 | -1.112 | -0.2601 | Yes |
| 144 | UACA |  |  | 5013 | -1.113 | -0.2521 | Yes |
| 145 | FEN1 |  |  | 5014 | -1.113 | -0.2441 | Yes |
| 146 | AURKB |  |  | 5016 | -1.118 | -0.2362 | Yes |
| 147 | TK1 |  |  | 5020 | -1.126 | -0.2287 | Yes |
| 148 | CDCA5 |  |  | 5023 | -1.133 | -0.2209 | Yes |
| 149 | TUBA1C |  |  | 5027 | -1.149 | -0.2132 | Yes |
| 150 | KIF2C |  |  | 5028 | -1.150 | -0.2049 | Yes |
| 151 | TOP2A |  |  | 5029 | -1.152 | -0.1967 | Yes |
| 152 | MYBL2 |  |  | 5041 | -1.172 | -0.1904 | Yes |
| 153 | TPX2 |  |  | 5049 | -1.184 | -0.1832 | Yes |
| 154 | CCNA2 |  |  | 5051 | -1.186 | -0.1749 | Yes |
| 155 | DEPDC1 |  |  | 5053 | -1.193 | -0.1665 | Yes |
| 156 | NDC80 |  |  | 5055 | -1.200 | -0.1580 | Yes |
| 157 | MT2A |  |  | 5061 | -1.216 | -0.1503 | Yes |
| 158 | ESCO2 |  |  | 5067 | -1.222 | -0.1424 | Yes |
| 159 | CDC20 |  |  | 5082 | -1.249 | -0.1362 | Yes |
| 160 | CENPK |  |  | 5091 | -1.267 | -0.1286 | Yes |
| 161 | UBE2C |  |  | 5106 | -1.314 | -0.1219 | Yes |
| 162 | CCDC34 |  |  | 5111 | -1.316 | -0.1132 | Yes |
| 163 | ZWINT |  |  | 5115 | -1.321 | -0.1043 | Yes |
| 164 | CKS1B |  |  | 5121 | -1.338 | -0.0956 | Yes |
| 165 | HJURP |  |  | 5125 | -1.344 | -0.0865 | Yes |
| 166 | RAD51AP1 |  |  | 5133 | -1.375 | -0.0780 | Yes |
| 167 | TYMS |  |  | 5156 | -1.477 | -0.0717 | Yes |
| 168 | CENPU |  |  | 5159 | -1.486 | -0.0614 | Yes |
| 169 | DIAPH3 |  |  | 5161 | -1.488 | -0.0509 | Yes |
| 170 | CDK1 |  |  | 5164 | -1.512 | -0.0404 | Yes |
| 171 | ANLN |  |  | 5183 | -1.577 | -0.0325 | Yes |
| 172 | CLSPN |  |  | 5202 | -1.663 | -0.0241 | Yes |
| 173 | PCLAF |  |  | 5209 | -1.698 | -0.0130 | Yes |
| 174 | RRM2 |  |  | 5232 | -1.875 | -0.0038 | Yes |
| 175 | HIST1H4C |  |  | 5249 | -2.081 | 0.0080 | Yes |
Table: GSEA details [plain text format]

  

Fig 2: DOROSZKO\_U3013MG\_CLUSTER\_2: Random ES distribution      
 Gene set null distribution of ES for **DOROSZKO\_U3013MG\_CLUSTER\_2**
